# Supplementary material for: Antibiotic Use in Broiler Poultry Farms in Kathmandu Valley of Nepal: Which Antibiotics and Why?
Source: Trop Med Infect Dis. 2021 Apr 5;6(2):47. doi: 10.3390/tropicalmed6020047 (PMC8167706; doi:10.3390/tropicalmed6020047)
Supplement: Supplementary file 1 [file tropicalmed-06-00047-s001.pdf]

Tropical Medicine and Infectious Disease

MDPI

Data collection form

Purpose: Antibiotic use in broiler poultry farms of Kathmandu valley.

Poultry Farm Number: ___________Study district: ___________Location: ___________

Poultry farm Name: ___________Date of Visit: ___________

Name of poultry in charge interviewed: ___________

Contact detail of in charge: ___________

Name of interviewer: ___________

1. **Type of antibiotics used (please tick one)**

Antibiotics Name	Yes	No
Tetracycline		
Neomycin		
Gentamycin		
Tylosin		
Sulphonamides		
Colistin sulphate		
Neomycin + Doxycycline		
Ciprofloxacin		
Livofloxacin		
Amoxicillin +Colistin		
Other _______________)		

2. **Reason of use of antibiotic (please tick one)**

Antibiotics Name	Prophylatic	Treat disease	Other (specify
Tetracycline			
Neomycin			
Gentamycin			
Tylosin			
Sulphonamides			
Colistin sulphate			
Neomycin + Doxycycline			
Ciprofloxacin			
Livofloxacin			
Amoxicillin +Colistin			
Others_______________)			
Others_______________)			
Others_______________)			

3. **Timing of antibiotic use for prophylaxis (Please tick one)**
(Skip this question if the farm use antibiotic only to treat disease)

Antibiotics Name	Continuous	Intermittent
Tetracycline		
Neomycin		
Gentamycin		
Tylosin		
Sulphonamides		
Colistin sulphate		
Neomycin + Doxycycline		
Ciprofloxacin		
Livofloxacin		
Amoxicillin +Colistin		
Other _______________)		

4. **Route of administration of Antibiotics(Please tick one)**

Antibiotics Name	Purpose Prophylactic, Treatment, Other)	In Feed	In water	Other Route
Tetracycline				
Neomycin				
Gentamycin				
Tylosin				
Sulphonamides				
Colistin sulphate				
Neomycin + Doxycycline				
Ciprofloxacin				
Livofloxacin				
Amoxicillin +Colistin				
Others_______________)				
Others_______________)				
Others_______________)				

5. **Total antibiotic consumed per month for all use (total Kg of antibiotics).(please indicate up to a maximum of the last 6 months from the month of visit)**

Months(2019)	In Kg
Month	
Month	
Month	
Month	
Month	
Month	

6. **Withdrawal period**

a. farmer is aware of the withdrawal period

1. Yes 2. No

b. If yes, number of days of withdrawal period prior to culling . ____________

(average number could be indicated)

7. **Purchase of Antibiotics**

a. with prescription	_
b. without prescription	_
c. Both	
